# Barriers and Opportunities for Cancer Clinical Trials in Low- and Middle-Income Countries

**DOI:** 10.1001/jamanetworkopen.2025.7733

**Published:** 2025-04-28

**Authors:** Linsey Eldridge, Nina R. Goodman, Amina Chtourou, Annette Galassi, Cecilia Monge, Mishka Kohli Cira, Paul C. Pearlman, Patrick J. Loehrer, Satish Gopal, Ophira Ginsburg

**Affiliations:** 1 Center for Global Health, National Cancer Institute, National Institutes of Health, Rockville, Maryland; 2 Office of Communications and Public Liaison, National Cancer Institute, National Institutes of Health, Rockville, Maryland; 3 Leidos Biomedical Research, Inc, Gaithersburg, Maryland; 4 Center for Cancer Research, National Cancer Institute, National Institutes of Health, Bethesda, Maryland; 5 Indiana University Melvin and Bren Simon Comprehensive Cancer Center, Indianapolis

## Abstract

**Importance:**

Clinical trials represent the gold standard to test the safety and efficacy of new or updated approaches to treatments that will inform quality cancer care. However, cancer trials enroll few patients in low- and middle-income countries (LMICs), are often led by investigators from high-income countries, and do not adequately reflect global disease burden or population diversity.

**Objective:**

To identify key challenges and strategies to advance contextually relevant, quality cancer trials in LMICs.

**Design, Setting, and Participants:**

The survey used in this survey study was available in English, Arabic, French, Portuguese, and Spanish and was conducted by the US National Cancer Institute from October 18 to December 22, 2023. Clinicians with experience in cancer therapeutic clinical trials in LMICs were eligible. The survey covered their professional background and views on challenges and strategies for improving clinical trial opportunities in LMICs. Analysis was performed from April 2 to August 26, 2024.

**Main Outcomes and Measures:**

Respondents were asked to rate 34 challenges by impact on their ability to conduct cancer trials using a 4-point Likert scale and 8 strategies by importance using a 5-point Likert scale. Descriptive statistics summarized participants’ backgrounds, challenges, and priorities.

**Results:**

Of 453 respondents who began the survey, a total of 223 (49%) were eligible for inclusion, and 131 of those (59%) completed the survey in full. Among the 133 respondents who provided gender data, 81 (61%) were male. In all, 107 of 130 respondents (82%) were affiliated with LMIC institutions, 65 of 223 (29%) were medical oncologists, and 52 of 133 (39%) were midcareer. Financial challenges were rated as the most impactful, with 133 of 170 respondents (78%) rating difficulty obtaining funding for investigator-initiated trials as having a large impact on ability to carry out a trial. Human capacity issues followed, with 105 of 192 respondents (55%) rating lack of dedicated research time as having a large impact. Increasing opportunities for funding and improving human capacity were reported as key strategies to advance capacity to conduct clinical trials in LMICs.

**Conclusions and Relevance:**

This survey study of clinicians with clinical trial experience in LMICs suggests that adequate funding and a well-trained research workforce are 2 predominant challenges to advancing cancer therapeutic clinical trials in LMICs. Understanding these obstacles can inform efforts to support cancer clinical trials that better reflect worldwide needs and diversity by prioritizing and sustaining research led by LMIC investigators.

## Key Points

**Question:**

What are the primary barriers to and most important strategies for conducting cancer therapeutic clinical trials in low- and middle-income countries (LMICs)?

**Findings:**

In this survey study of 223 clinicians with cancer therapeutic clinical trial experience in LMICs, the most impactful barriers were lack of funding for investigator-initiated trials and lack of dedicated research time. Increasing funding opportunities and improving human capacity were viewed as the most important strategies to improve opportunities for LMIC-led cancer trials.

**Meaning:**

The findings suggest that for clinical trials to be LMIC led and relevant, there is need for substantive, strategic investments in cancer clinical trial ecosystems in LMICs.

## Teaser

This survey study identifies key barriers to and strategies for conducting cancer therapeutic clinical trials led by investigators in low- and middle-income countries.

## Introduction

Clinical trials that test the safety and therapeutic benefit of cancer drugs and other treatments are essential for developing new standards of care for patients living with cancer.^1^ Benefits of clinical trial participation include access to new therapies, close monitoring and support from the clinical trial team, potentially better survival compared with nonparticipants, and opportunities to contribute to the knowledge base that benefits other patients.^2,3,4^

Despite the importance of clinical trials, few patients enroll in therapeutic clinical trials,^5^ and those that do tend to be younger with fewer comorbidities and are less racially and ethnically diverse than patients who do not enroll in trials, limiting generalizability.^6,7,8^ In low- and middle-income countries (LMICs), patients and investigators often do not have opportunities to participate in cancer therapeutic clinical trials (referred to as *cancer trials* hereafter) due to lack of funding and training, inadequate infrastructure, and a limited clinical research culture.^9,10,11^ Despite the globalization of industry-sponsored oncology trials,^12^ just under one-third of high-income country (HIC)–led cancer trials between 2014 and 2017 enrolled patients from LMICs.^13^ Trials that do enroll patients from LMICs are often led by investigators from HICs, with only 8% of phase 3 oncology randomized clinical trials led by investigators from LMICs.^14^ As a result, cancer trials often do not reflect global disease burden, population diversity, and health system realities, limiting the worldwide generalizability and applicability of results.^15,16^

Lack of representation and generalizability is especially concerning in light of the increasing burden of cancer in LMICs, where 70% of cancer deaths occur.^17^ To address the escalating worldwide cancer burden, there is a critical need for research funders and decision-makers to support evidence generation that can inform practice and policies in LMICs, including high-quality, context-relevant cancer trials that are led by investigators in LMICs and are patient-centered with clinically meaningful end points.

To better understand the challenges related to conducting cancer trials in LMICs and to identify potential strategies and priorities to improve opportunities for LMIC-based investigators to conduct cancer trials, the US National Cancer Institute (NCI) Center for Global Health surveyed clinicians with experience conducting cancer trials in LMICs. This article describes the findings from the survey and discusses their implications for the oncology research community.

## Methods

### Survey Design

The survey used in this survey study was developed by a team at the NCI (including all of us) with expertise in clinical trials, global oncology, and survey design and methods. An online questionnaire was developed using the Qualtrics online survey platform. The survey content was based on findings from formative research and engagement activities. These included an NCI-published request for information in 2023, stakeholder engagement meetings held at the World Cancer Congress in 2022 and the American Society of Clinical Oncology 2023 annual meeting, and a series of key informant interviews. The National Institutes of Health institutional review board determined that ethical review was not required for this survey study because it was not human participants research; as such, informed consent was not required. This study followed the American Association for Public Opinion Research (AAPOR) reporting guideline for survey studies.

The key informant interviews were held with 14 thought leaders with experience conducting cancer trials in LMICs, representing diverse specialties and geographic locations at different career stages. These thought leaders participated in a 1-hour, semistructured interview via a cloud-based videoconferencing platform between October 11, 2022, and January 4, 2023. Interviewees were asked about their opinions regarding top challenges to implementing cancer trials in LMICs and strategies to address these challenges. Interviews were recorded and transcribed, then double coded using Dedoose, a web-based application for analyzing qualitative research data. The top challenges and strategies were used to inform development of the survey questions.

The survey was piloted and revised based on feedback. The final survey consisted of 5 sections with 30 questions and took 10 to 15 minutes to complete. The first 2 questions determined eligibility. Although the survey was anonymous, respondents were given the option to voluntarily provide their name and email address. The survey was available in English, Arabic, French, Portuguese, and Spanish to improve accessibility and diversity of geographic regions.

The 5 areas assessed included (1) demographic information and professional experience, (2) clinical research experience, (3) challenges to conducting clinical trials, (4) strategies to overcome challenges, and (5) proposed clinical research priorities (eMethods in Supplement 1). A total of 34 challenges organized into 8 categories were rated using a 4-point Likert scale by level of impact on ability to carry out a clinical trial (1 indicating “no impact” and 4, “large impact”). Eight strategies to increase opportunities to conduct clinical trials were rated by level of importance using a 5-point Likert scale (1 indicating “not at all important” and 5, “extremely important”). There were free-text response options for additional comments. Clinicians without relevant experience as per survey eligibility criteria but who reported interest in cancer trials were then asked about the primary barriers they experienced that prevented them from becoming involved in trials.

### Study Population

The sample size for this survey was calculated to achieve a margin of error of 5% with a 95% CI. Based on standard sample size estimation formulas for proportions, a total of 380 responses were determined to be necessary to ensure results within the specified margin of error. Clinicians with experience conducting at least 1 cancer trial with at least 1 recruitment site in an LMIC, based on the 2023 World Bank income classification inclusive of low-, lower-middle, and upper-middle income, were eligible for inclusion in this survey.

The sampling frame was a global network of cancer clinicians and researchers derived from 2 sources: membership in national and regional oncology organizations and identification as a principal investigator in cancer treatment trials in at least 1 LMIC between 2020 and 2023. Principal investigators were identified by 3 sources: the Global Oncology Survey of NCI-Designated Cancer Centers,^18^ the International Clinical Trials Registry, and ClinicalTrials.gov.

The web-based survey was distributed using a hierarchical snowball method. Invitations to distribute the survey were sent via email to oncology contacts at 160 organizations (eTable 1 in Supplement 1), and invitations to complete and subsequently distribute the survey were sent to 660 individuals. Three reminders were sent to each organization contact. One reminder was sent to individuals. The survey was also promoted on social media. Survey responses were collected from October 18 to December 22, 2023. No compensation for participation was given.

### Statistical Analysis

After excluding responses not meeting eligibility criteria, survey responses were exported into Microsoft Excel, version 2407. All responses to individual survey items were included in the analysis even if the entire survey was not completed, resulting in varying denominators across survey items.

Analysis was performed from April 2 to August 26, 2024. Descriptive statistics were used to describe demographics, research experience, perceived challenges and strategies, and opinions regarding research priorities. For bivariate analyses of perceived challenges, Likert scale impact responses of “large” and “moderate” were grouped together, as were “no” and “slight,” to account for small cell sizes. Categorical variables were compared using the Fisher exact test and χ^2^ test. A 2-sided *P* value less than 0.05 was used to determine statistical significance. Descriptive statistics were completed in Microsoft Excel, version 2407, and statistical analyses were performed using SAS, version 9.4 (SAS Institute Inc).

For the qualitative analysis, free-text responses regarding challenges and strategies were imported into Microsoft Excel, version 2407, for coding and analysis. The coding scheme included 16 high-level categories (8 challenges and 8 strategies) provided in the survey and an “other” category for responses that did not fit a preexisting category. Three team members (L.E., A.G., M.K.C.) independently coded all responses, and discrepancies were resolved with discussion. Themes were extracted from the “other” responses and were systematically applied to all responses, including those previously categorized. Responses tended to be brief, containing a single idea aligned with the high-level categories.

## Results

### Professional Background and Research Experience

**Figure 1.**
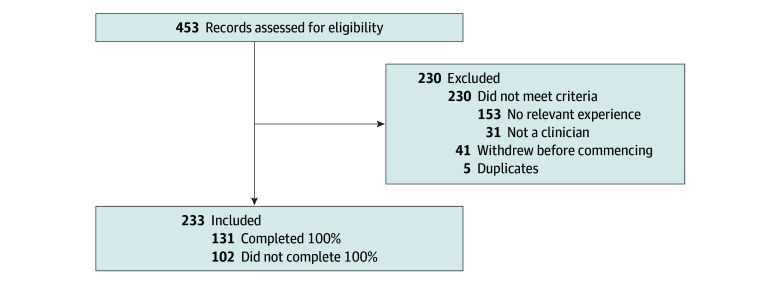
Respondent Flowchart

Overall, 453 respondents began the survey; 31 were excluded because they were not clinicians, 153 were excluded because they did not have relevant experience, 41 were eligible but were excluded because they withdrew before commencing the survey, and 5 duplicates were removed (the earliest duplicate response was kept and supplemented if additional information was provided in the later response). The final study cohort consisted of 223 respondents (49% of those who began the survey), of whom 131 (59%) completed the survey in full, with a median completion rate of 100% (IQR, 41.5%-100%) (Figure 1).

Demographics, professional background, and research experience are summarized in Table 1. Among the 133 respondents who provided gender data, 50 (38%) were female; 81 (61%) were male; none were transgender, nonbinary, or another gender; and 2 (2%) declined to answer. Most respondents were midcareer (52 of 133 [39%]) or late career (49 of 133 [37%]). Respondents reported 26 different specialties, with medical oncology (65 of 223 [29%]) and clinical oncology (39 of 223 [17%]) being most common. Over half (115 of 220 [52%]) worked in academic settings. Respondents reported being affiliated with institutions in 41 countries across 7 regions (eTable 2 in Supplement 1). The most common region was sub-Saharan Africa (39 of 130 [30%]), while the most common country was India (17 of 130 [13%]). Most respondents (107 of 130 [82%]) were affiliated with institutions in LMICs.

**Table 1.** Demographic Characteristics and Professional Experience of Respondents

Characteristic	Respondents, No./total No. (%)^a^
Gender	
Female	50/133 (38)
Male	81/133 (61)
Transgender, nonbinary, or another gender	0
Declined to answer	2/133 (2)
Career stage^b^	
Student	0
Trainee	3/133 (2)
Early career	28/133 (21)
Midcareer	52/133 (39)
Late career	49/133 (37)
Other	1/133 (1)
Specialty	
Clinical oncology	39/223 (17)
General practice	9/223 (4)
Gynecology-oncology	11/223 (5)
Medical oncology	65/223 (29)
Nursing	13/223 (6)
Pediatric oncology	19/223 (9)
Radiotherapy	10/223 (5)
Researcher	8/223 (4)
Surgery	17/223 (8)
Other^c^	32/223 (14)
Work setting	
Academic, such as a college or university	115/220 (52)
Public hospital, health center, or clinic	91/220 (41)
Private hospital, health center, or clinic	66/220 (30)
Nonprofit organization	31/220 (14)
Industry or pharmaceutical company	3/220 (1)
Government, such as a ministry of health	16/220 (7)
Other^d^	1/220 (1)
Country income level of primary affiliation^e^	
High	23/130 (18)
Upper middle	46/130 (35)
Lower middle	51/130 (39)
Low	10/130 (8)
Region of primary affiliation	
East Asia and Pacific	12/130 (9)
Europe and Central Asia	8/130 (6)
Latin America and the Caribbean	34/130 (26)
Middle East and North Africa	4/130 (3)
North America	15/130 (12)
South Asia	18/130 (14)
Sub-Saharan Africa	39/130 (30)
Research experience	
Clinical trial role	
Principal investigator	101/139 (73)
Coinvestigator	100/139 (72)
Site principal investigator	71/139 (51)
Other^f^	13/139 (9)
Cancer site	
Breast	74/139 (53)
Cervical	32/139 (23)
Colorectal	27/139 (19)
Esophageal	20/139 (14)
HIV-associated	18/139 (13)
Leukemia	13/139 (9)
Lip or oral cavity	10/139 (7)
Liver	9/139 (6)
Lung	36/139 (26)
Lymphoma	21/139 (15)
Pancreas	9/139 (6)
Pediatric	18/139 (13)
Prostate	23/139 (17)
Stomach	21/139 (15)
Other^g^	35/139 (25)
Trial phase	
1	7/138 (5)
2	29/138 (21)
3	84/138 (61)
4	18/138 (13)
Proportion of trials recruiting most or all patients from an LMIC site, %	
<25	40/136 (29)
25-50	14/136 (10)
51-75	7/136 (5)
76-100	40/136 (29)
Unsure	35/136 (26)
Funding source	
Industry	97/134 (72)
Government	67/134 (50)
Academic	82/134 (61)
Other^h^	17/134 (13)
Unspecified	17/134 (13)

Abbreviation: LMIC, low- or middle-income country.

^a^ Percentages may not sum to 100% due to multiple categories selected. Partial responses were included, resulting in varying denominators for each domain. Percentages were calculated based on those who responded.

^b^ Early career was less than 10 years; midcareer, 10 to 20 years; late career, 20 or more years; and other, unspecified.

^c^ Other specialties included hematology (5 [2%]), pathology (4 [2%]), epidemiology (3 [1%]), internal medicine (3 [1%]), pharmacy (3 [1%]), gastroenterology (2 [1%]), pharmacology (2 [1%]), anesthesiology (1 [0%]), clinical officer oncology (1 [0%]), coordinator (1 [0%]), infectious disease (1 [0%]), medical psychology (1 [0%]), microbiology (1 [0%]), obstetrics and gynecology (1 [0%]), palliative care (1 [0%]), psychology (1 [0%]), and radiology (1 [0%]).

^d^ Community-based organization.

^e^ Based on 2023 World Bank country income group classifications.

^f^ Other clinical trial roles included study coordinator (4 [3%]), nurse (2 [1%]), patient accrual (2 [1%]), consultant or mentor (1 [1%]), mentor (1 [1%]), medical monitor (1 [1%]), pharmacist (1 [1%]), psychosocial care (1 [1%]), and study physician (1 [1%]). One person entered 2 roles (study coordinator and nurse).

^g^ Other cancer sites included non–site specific (8 [6%]), head and neck (5 [4%]), nervous system (3 [2%]), bladder (3 [2%]), endometrial (3 [2%]), melanoma (3 [2%]), kidney (3 [2%]), ovary (2 [1%]), Kaposi sarcoma (2 [1%]), liver (2 [1%]), neuroendocrine (2 [1%]), myeloma (1 [1%]), sarcoma (1 [1%]), urinary system (1 [1%]), and anal (1 [%]).

^h^ Other funding sources included nonprofit or nongovernmental organizations (11 [8%]), grants (3 [2%]), private (2 [1%]), independent research group (1 [1%]), professional society (1 [1%]), and self-funded (1 [1%]).

Most respondents (101 of 139 [73%]) reported having been a principal investigator on at least 1 cancer trial, and 84 of 138 (61%) reported that most of their trials were phase 3 studies. The most common cancer sites reported were breast (74 of 139 [53%]), lung (36 of 139 [26%]), and cervical (32 of 139 [23%]). Nearly three-quarters of respondents (97 of 134 [72%]) received some funding from industry.

### Perceived Challenges

Respondents reported experiencing many barriers to conducting clinical trials in LMICs. Among the 34 challenges provided, 24 (71%) were rated as having a large impact on ability to conduct cancer trials by 90% or more of respondents. The barrier rated as having the greatest impact was difficulty obtaining funding for investigator-initiated trials, with 133 of 170 respondents (78%) rating this as having a large impact. Overall, financial challenges were rated highest by level of impact, followed by human capacity challenges, with 105 of 192 respondents (55%) rating lack of dedicated research time as having a large impact. While lack of research training did not emerge as a top-rated barrier by all respondents, trainees and early-career respondents were more likely to rate it as having a moderate or large impact (29 of 32 [91%]) compared with midcareer or late-career respondents (67 of 101 [66%]) (*P* = .007). Other prominent barriers included difficulties accessing care, lack of access to drugs or products, and lack of state political will for cancer trials. The full list of challenges is provided in Table 2.

**Table 2.** Challenges in Conducting Cancer Clinical Trials in LMICs

Challenge	Total respondents, No.	Responses, No. (%)^a^			
		No impact	Slight impact	Moderate impact	Large impact
**Human capacity**					
Lack of health care provider awareness of trials	192	10 (5)	43 (22)	79 (41)	60 (31)
Lack of mentorship	193	12 (6)	45 (23)	75 (39)	61 (32)
Lack of research training	194	17 (9)	34 (18)	71 (37)	72 (37)
Personnel shortage	194	12 (6)	23 (12)	76 (39)	83 (43)
Competing priorities	193	7 (4)	28 (15)	66 (34)	92 (48)
Lack of dedicated research time	192	6 (3)	22 (12)	59 (31)	105 (55)
**Infrastructure and resources**					
Lack of access to drugs or products	182	15 (8)	26 (14)	54 (30)	87 (48)
Equipment shortages	183	16 (9)	31 (17)	67 (37)	69 (38)
Difficulties with data management	183	16 (9)	38 (21)	73 (40)	56 (31)
Space and storage shortage	183	23 (13)	53 (29)	70 (38)	37 (20)
Insufficient biobanking	183	18 (10)	38 (21)	65 (36)	62 (34)
Insufficient diagnosis	182	21 (12)	51 (28)	43 (24)	67 (37)
**Ethical and regulatory**					
Decision delays	177	14 (8)	37 (21)	61 (35)	65 (37)
Burdensome procedures	177	14 (8)	34 (19)	66 (37)	63 (36)
Lack of trained regulatory authorities	177	61 (35)	56 (32)	25 (14)	35 (20)
**Financial**					
Difficulty obtaining funding for investigator-initiated trials	170	4 (2)	5 (3)	28 (17)	133 (78)
Difficulty obtaining funding in general	168	5 (3)	7 (4)	33 (20)	123 (73)
Complex grant application or funding process	169	5 (3)	9 (5)	51 (30)	104 (62)
Excessive trial costs	170	13 (8)	15 (9)	53 (31)	89 (52)
Lack of interest by pharmaceutical companies	170	15 (9)	20 (12)	58 (34)	77 (45)
**Administrative**					
Lack of institutional experience with trial management	167	20 (12)	35 (21)	61 (37)	51 (31)
Lack of institutional support for research	167	18 (11)	33 (20)	60 (36)	56 (34)
**Health care or sociopolitical**					
Fragmented health care system	162	9 (6)	31 (19)	54 (33)	68 (42)
Coordination of efforts to implement cancer clinical trials	161	5 (3)	28 (17)	66 (41)	62 (39)
Lack of government political will	161	14 (9)	25 (16)	45 (28)	77 (48)
Difficulties identifying collaborators or partners	161	12 (8)	28 (17)	66 (41)	55 (34)
**Trial design**					
Irrelevant study questions	161	33 (21)	61 (38)	41 (26)	26 (16)
Inappropriate standard therapy arm	161	33 (21)	43 (27)	52 (32)	33 (21)
Increasing complexity of trials	161	18 (11)	34 (21)	64 (40)	45 (28)
**Patient enrollment**					
Lack of insurance coverage	157	20 (13)	25 (16)	38 (24)	74 (47)
Patient difficulties with accessing care	157	11 (7)	22 (14)	45 (29)	79 (50)
Restrictive eligibility	156	15 (10)	40 (26)	49 (31)	52 (33)
Distrust of clinical research or medical practitioners	157	21 (13)	51 (33)	46 (29)	39 (25)
Lack of clinical trial awareness	155	13 (8)	36 (23)	41 (27)	65 (42)

Abbreviation: LMIC, low- or middle-income country.

In the free-text fields, respondents reiterated challenges already provided. Most were related to financial challenges (mentioned in 85 of 357 responses [24%] and by 53 of the 112 participants [47%] who provided free-text responses), human capacity challenges (74 of 357 responses [21%] and 50 of 112 participants [45%]), and challenges related to patient recruitment or engagement (68 of 357 responses [19%] and 45 of 112 participants [40%]).

Respondents also identified other challenges not provided in the survey. Most commonly, respondents described insufficient salary and funding for personnel, an unsupportive research environment, and collaboration challenges, such as between institutions in LMICs and HICs or among colleagues at their institution.

Respondents provided additional details about barriers in the free-text responses. For example, human capacity challenges included insufficient staff to handle administrative tasks, such as project management and grant administration. Regarding financial challenges, respondents added that current funding processes do not allow for advance payment for personnel and infrastructure necessary to begin a clinical trial. Respondents also provided additional context about the setting in which they are attempting to conduct clinical trials. Representative examples of this context are below.

Most patients in my country pay out of pocket for cancer care. Some can’t afford diagnostic tests…to enable them being selected for screening…Most patients travel far distances to access care in cancer centers and sometimes need support for transportation and accommodation…These are mostly not covered by clinical trials. Majority of our patients present late, especially with locally advanced diseases, and may not be eligible for studies.—Male respondent, midcareer, clinical oncologist, sub-Saharan Africa

There are so many patients that need to be seen that the idea of protected time for research is often a bit absurd. It may need a full time other person who is actually the one running the clinical trial. If the hematologist-oncologist attempts to take on the clinical trial tasks, you may be setting yourself up for failure due to lack of time, as the clinicians are trying to make enough money to keep living a higher standard of living and will also use the clinical trial money to do the same, even if they don’t have time.—Male respondent, early career, hematology, sub-Saharan Africa

There is lack of awareness of clinical research and a general mistrust of research among participants, compounded by illiteracy, social barriers, difficulties in accessing health care, and lack of familiarity with technology. For researchers, clinical work is prioritized over research, and huge patient volumes and lack of protected research time are challenges.—Female respondent, late career, anesthesiologist, South Asia

Lack of investigator-initiated study support to address the contextual priority problems that would receive sociopolitical support. For example, cervical cancer is a socially devastating disease. Clinical trials in this area and that of breast and prostate cancer, even colon or rectal cancer, would receive political support. A country dialogue on the priority cancers to be addressed in clinical trials will be necessary.—Female respondent, trainee, epidemiology, sub-Saharan Africa

Respondents without experience and with interest in conducting a cancer trial in an LMIC were asked about the primary barriers preventing them from being involved. The most reported barrier among these 139 respondents was lack of funding (83 [60%]) (eTable 3 in Supplement 1).

### Perceived Strategies and Proposed Priorities

**Figure 2.**
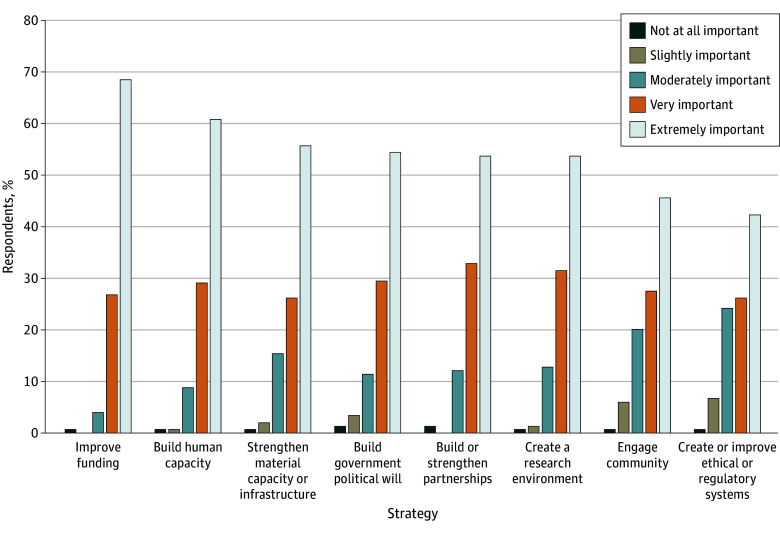
Strategies for Conducting Cancer Clinical Trials in Low- and Middle-Income Countries, Rated by Level of Importance on a Likert Scale

Respondents agreed that many of the strategies listed in the survey are important for improving opportunities for clinical trials in LMICs. Of the 8 strategies, 6 were rated as very or extremely important by at least 80% of respondents. The most important strategy was improving funding (102 of 149 [68%] rated this as extremely important), followed by building human capacity (90 of 148 [61%] rated this as extremely important). The full list of strategies is shown in Figure 2.

The most frequently reported strategies highlighted in the free-text responses were related to building human capacity (22 of 48 responses [46%]), building state political will (14 of 48 responses [29%]), and strengthening material capacity or infrastructure (10 of 48 responses [21%]). Additional strategies in the free-text responses include prioritizing locally relevant scientific questions, improving care across the cancer control continuum, and strengthening health systems in LMICs.

The most common types of trials that respondents recommended prioritizing in LMICs were dose deescalation, efficiency, or pragmatic trials (120 of 145 [83%]), followed by late-phase studies (109 of 145 [75%]). Respondents also recommended that trials be multinational with sites in both HICs and LMICS (98 of 145 [68%]) (Table 3).

**Table 3.** Characteristics of Proposed Cancer Therapeutic Clinical Trials in LMICs

Characteristic	Trials, No. (%) (n = 145)^a^
Trial type	
Early-phase development (phase 1-2)	68 (47)
Late-phase development (phase 3-4)	109 (75)
Deescalation, efficiency, or pragmatic	120 (83)
Other^b^	15 (10)
Geographic focus	
Multinational trials in HICs and LMICs	98 (68)
Multinational trials in LMICs only	37 (26)
Trials restricted to 1 LMIC	7 (5)
Other^c^	3 (2)

Abbreviations: HIC, high-income country; LMIC, low- or middle-income country.

^a^ Percentages may not sum to 100% due to multiple categories selected. Partial responses were included, resulting in varying denominators for each survey question. Percentages were calculated based on those who responded.

^b^ Other trial types included prevention (2 [1%]), quality of life (2 [1%]), locally relevant (2 [1%]), hybrid (1 [1%]), cost-effectiveness (1 [1%]), and resource stratified (1 [1%]).

^c^ Other geographic foci included all of the above (2 [1%]), multinational trials in LMICs only (1 [1%]), and multinational trials in HICs and LMICs (1 [1%]).

## Discussion

Clinical trials are the gold standard for assessing safety and efficacy of therapeutic interventions and can help advance equitable cancer care. Despite the growing burden of cancer, patients and investigators from LMICs are insufficiently represented in the current clinical trial landscape. Trials that do recruit patients in LMICs are often designed and led by investigators from HICs and often do not reflect the local context and expertise, cancer burden, or patient needs. In this study, we surveyed cancer clinical trialists working in LMICs to explore key challenges and potential solutions to address these gaps.

First, many challenges impede opportunities for clinical trials in LMICs. All 34 listed challenges were perceived by respondents as having some level of impact on the ability to conduct trials. In LMICs, the most significant barrier was obtaining funding for investigator-initiated studies. Most respondents reported receiving industry funding for their trials. This aligns with previously reported findings that most cancer clinical trials in LMICs are funded by industry and led by investigators in HICs.^13,14,19^

Human capacity–related barriers, specifically a lack of protected research time, were the second-highest-rated challenges. This finding is in line with studies revealing severe cancer workforce shortages and substantially higher patient volume.^20,21^ Trainees and early-career respondents identified lack of training and mentorship as impactful barriers. Training is vital to ensure research teams consist of skilled investigators, nurses, statisticians, and trial coordinators to help balance administrative tasks with patient care. The success of the International Collaboration for Research Methods Development in Oncology workshop demonstrates this importance.^22^

Third, late-phase clinical trials and trials designed to address contextually appropriate questions that will have clinical implications were a priority among clinicians involved in LMIC trials. Over three-quarters of respondents (83%) selected deescalation, efficiency, or pragmatic trials as a priority. In recent years, there has been a substantial increase in the number of oncology trials investigating novel therapies using surrogate end points as industry-funded trials have surpassed investigator-initiated trials.^17^ Despite this, many of the medications that continue to be rated most important by oncologists received US Food and Drug Administration approval before 2000, highlighting a continued need to improve worldwide access and applicability of existing therapies.^17,23^ Our findings are consistent with emerging principles, guided by groups such as Common Sense Oncology, toward clinically meaningful trials with patient-centered outcomes.^24^ Such efforts are motivated by the need to improve the global public health return on cancer clinical trial investments given the growing complexity and costs of oncology trials and to avoid yielding prohibitively expensive interventions with marginal benefits.^25^

In addition, strengthening the partnership between institutions in HICs and LMICs was a priority and strategy for addressing barriers. Such partnerships can enhance clinical trial capacity by fostering bidirectional knowledge exchange, leveraging resources, and building local expertise. Respondents suggested a network of multinational trials with sites in both HICs and LMICs. However, collaboration challenges between HIC and LMIC institutions, such as lack of trust and cultural differences, were identified as a challenge. While collaborations can provide essential support in the form of funding and capacity building, global partnerships have often been imbued with inequity, favoring researchers in HICs.^26,27^ Investigators in LMICs are often viewed as implementers in the field, with insignificant involvement in research development, and they do not receive appropriate credit.^28,29^ Cross-country income partnerships should be designed to ensure that LMIC institutions are not passive recipients of knowledge but active and equal leaders in the research process. Fostering equitable partnerships can drive more inclusive and context-specific research agendas, enhance the relevance and applicability of findings, and ultimately lead to more equitable, high-quality cancer care that benefits patients worldwide.^30,31^

New investments or reprioritizations where relevant should be country led and can address a country’s context in terms of the cancer burden, population, and the largest gaps and greatest needs in infrastructure, personnel, and overall systems readiness. Countries with limited cancer research capacity that want to prioritize strengthening the cancer clinical trials infrastructure can consider leveraging existing clinical trials infrastructure and personnel, for example, in settings with well-established clinical trials ecosystems for infectious diseases. Strengthening capacity will also ensure researchers in LMICs become leaders who are competitive to receive funding directly. Over time, it will be essential for countries to invest in establishing or strengthening centers with dedicated clinical trial capacity to optimize evidence-informed, high-quality patient care. Sustaining research capacity will require diversifying funding sources, including government funding, by building political will, promoting a culture of research, and engaging the broader community. A long-term research-strengthening strategy fits well as a component of a country’s cancer policy that serves to guide the planning, financing, and provision of equitable access to affordable, quality cancer care for a country’s population.

### Limitations

To our knowledge, this is the first survey focusing on cancer therapeutic clinical trials in LMICs with participation from all worldwide regions. Our study has several important limitations. First, the snowball sampling method has potential for sampling bias. Second, to protect the privacy and anonymity of respondents, we did not collect personally identifiable information except when volunteered, which may have resulted in duplicate responses. Third, the exclusion criteria left out the perspective of nonclinicians. The NCI is currently leading additional engagement activities, including focus group discussions with patient advocates in LMICs about patient engagement in clinical trials. Fourth, we did not reach our planned sample size of 380 responses. Our study was not adequately powered to perform subanalyses that might reveal differences between groups, such as by gender or region.

## Conclusions

This survey study of clinicians with clinical trial experience in LMICs found that adequate funding and a well-trained research workforce were 2 predominant challenges to advancing cancer therapeutic clinical trials in LMICs. The findings suggest that for clinical trials to be LMIC led and relevant, there is need for substantive, strategic investments in cancer clinical trial ecosystems in LMICs. While the challenges identified in this survey are not unique to LMICs, the resource constraints, health care infrastructures, and socioeconomic factors in LMICs have severely limited opportunities available for cancer trials and require tailored solutions.

## Notes

Supplement 1. **eTable 1.** Distribution Networks **eTable 2.** Survey Responses by Respondents’ Country of Affiliation **eTable 3.** Barriers to Cancer Therapeutic Clinical Trial Involvement by Excluded Respondents **eMethods.** Survey on Cancer Therapeutic Clinical Trials in Low- and Middle-Income Countries

Supplement 2.
Data Sharing Statement
