# Supplementary material for: Barriers and Opportunities for Cancer Clinical Trials in Low- and Middle-Income Countries
Source: JAMA Netw Open. 2025 Apr 28;8(4):e257733. doi: 10.1001/jamanetworkopen.2025.7733 (PMC12038506; doi:10.1001/jamanetworkopen.2025.7733)
Supplement: Supplement 1. — eTable 1. Distribution Networks eTable 2. Survey Responses by Respondents’ Country of Affiliation eTable 3. Barriers to Cancer Therapeutic Clinical Trial Involvement by Excluded Respondents eMethods. Survey on Cancer Therapeutic Clinical Trials in Low- and Middle-Income Countries [file jamanetwopen-e257733-s001.pdf]

Supplementary Online Content

Eldridge L, Goodman NR, Chtourou A, et al. Barriers and opportunities for cancer clinical trials in low- and middle-income countries. *JAMA Netw Open*. 2025;8(4):e257733. doi:10.1001/jamanetworkopen.2025.7733

eTable 1. Distribution Networks

eTable 2. Survey Responses by Respondents' Country of Affiliation

eTable 3. Barriers to Cancer Therapeutic Clinical Trial Involvement by Excluded Respondents

eMethods. Survey on Cancer Therapeutic Clinical Trials in Low- and Middle-Income Countries

This supplementary material has been provided by the authors to give readers additional information about their work.

© 2025 Eldridge L et al. *JAMA Network Open*.

eTable1 – Distribution Networks

Organization
Abramson Cancer Center
Advanced Clinical Research Training Institute in Latin America-American Society of Hematology
Africa Cancer Research and Control ECHO
African Academy of Sciences
African Organisation for Research and Training in Cancer (AORTIC)
African Population Health and Research Center
Albert Einstein Cancer Center
Alvin J. Siteman Cancer Center - Washington University School of Medicine and Barnes-Jewish Hospital
American Association for Cancer Research
American Cancer Society (ACS)
American Society of Clinical Oncology (ASCO)
American Society of Clinical Pathology (ASCP)
AORTIC Nursing
Arizona Cancer Center
Asian Fund for Cancer Research Limited
Asian Oncology Nursing Society
Asociación Argentina de Oncología Clínica
Asociación Colombiana de Hematología y Oncología
Asociación Colombiana de Hematología y Oncología Pediátrica
Barbara Ann Karmanos Cancer Center
BioVentures for Global Health
Breast Health Global Initiative (BHGI)
Canadian Partnership Against Cancer
Cancer Council of the Pacific Islands
Case Comprehensive Cancer Center
Centers for Disease Control and Prevention USA (CDC)
Chao Family Comprehensive Cancer Center
Chinese National Cancer Center
City Cancer Challenge
City of Hope Comprehensive Cancer Center
Coalition of Implementation Research in Global Oncology
Consortium of Universities for Global Health
DAC Trials-Gates Foundation
Dan L. Duncan Comprehensive Cancer Center
Dana-Farber/Harvard Cancer Center
Dharmais National Cancer Center, Indonesia
Division of Cancer Prevention
**Organization**
Duke Cancer Institute

© 2025 Eldridge L et al. *JAMA Network Open*.

ECHO ELA
European Society for Medical Oncology
Extension for Community Healthcare Outcomes (Project ECHO)
Fogarty International Center
Fox Chase Cancer Center
Fred & Pamela Buffett Cancer Center
Fred Hutchinson/University of Washington Cancer Consortium
Georgetown Lombardi Comprehensive Cancer Center
Global Focus on Cancer
Global Forum of Cancer Surgeons/Society of Surgical Oncology
Global Research Nurses
H. Lee Moffitt Cancer Center and Research Institute
Harold C. Simmons Cancer Center
Herbert Irving Comprehensive Cancer Center
Holden Comprehensive Cancer Center/University of Iowa
Hollings Cancer Center
Huntsman Cancer Institute - University of Utah
IAEA PACT
Indiana University Melvin & Bren Simon Cancer Center
Indonesian Oncology Nurses Association
International Agency for Research on Cancer (IARC)
International Association of Clinical Research Nurses
International Atomic Energy Agency (IAEA)
International Cancer Research Partnership (ICRP)
International Cancer Screening Network
International Gynecologic Cancer Society
International Network for Cancer Training and Research
International Rare Cancer Initiative
International Society of Gastrointestinal Oncology
International Society of Nurses in Cancer Care
International Society of Paediatric Oncology - Africa
International Society of Paediatric Oncology - Latin America
Intravenous and Chemotherapy Association of Thailand
Jonsson Comprehensive Cancer Center
Kings College London
Latin American and Caribbean Society of Medical Oncology (SLACOM)
Laura and Isaac Perlmutter Cancer Center at NYU Langone
Lebanese Society of Medical Oncology
**Organization**
Maria Sklodowska Curie National Research Institute of Oncology
Markey Cancer Center
Masonic Cancer Center
Massey Cancer Center

© 2025 Eldridge L et al. *JAMA Network Open*.

Mayo Clinic Cancer Center
Mays Cancer Center
Memorial Sloan Kettering Cancer Center
Moores Comprehensive Cancer Center
MRC Clinical Trials Unit (MRC CTU) at UCL
National Cancer Instiute of Kenya
National Clinical Research Center for Cancer, Tianjin Cancer Institute
National Comprehensive Cancer Network (NCCN)
NCTN - Alliance
NCTN - Canadian Network Group
NHLBI-CTRIS
NIH/NCI Center for Cancer Research
NIH/NCI Center for Cancer Training
NIH/NCI Division of Cancer Control and Population Sciences
NIH/NCI Division of Cancer Epidemiology and Genetics
NIH/NCI Division of Cancer Treatment and Diagnosis
NIH/NCI Office of Advocacy Relations
NIH/NCI Office of HIV and AIDS Malignancy
Norris Cotton Cancer Center
OHSU Knight Cancer Institute
Oncology Nursing Society - US
O'Neal Comprehensive Cancer Center at UAB
ONS Clinical Trials Community
ONS Global Onc Community
Pan-American Health Organisation (PAHO)
Philippine Oncology Nurse Association, INC
Philippine Radiation Oncology Society
Philippine Society of Medical Oncology
Princess Margaret Cancer Center
Purdue University Center for Cancer Research
Queen's University Department of Oncology & Global Oncology Program
Red de Institutos Nacionales de Cáncer (RINC)
Rising Tide Foundation
Robert H. Lurie Comprehensive Cancer Center
Roswell Park Cancer Institute
**Organization**
Rutgers Cancer Institute of New Jersey
Sidney Kimmel Cancer Center at Thomas Jefferson University
Sidney Kimmel Comprehensive Cancer Center
Sociedad Boliviana de Cancerología
Sociedad Chilena de Oncología Médica
Sociedad de Oncología Médica y Pediátrica del Uruguay
Sociedad Peruana de Oncología Medica

© 2025 Eldridge L et al. *JAMA Network Open*.

Sociedade Brasileira de Oncologia Clínica
Society of Gynecologic Oncologists of the Philippines
St. Jude Children's Research Hospital
Stanford Cancer Institute
Stephenson Cancer Center
Surgical Oncology Society of the Philippines
Sylvester Comprehensive Cancer Center
The Middle Eastern Association for Cancer Research
The Ohio State University Comprehensive Cancer Center-Artuhr G. James Cancer Hospital and Richard J. Solove Research Institute (OSUCCC-The James)
The University of Virginia Cancer Center
Tiawan Oncology Nursing Society
Tisch Cancer Institute
Two Worlds Cancer Collaboration
UC Davis Comprehensive Cancer Center
UCSF Helen Diller Family Comprehensive Cancer Center
Uganda Cancer Institute
UNC Lineberger Comprehensive Cancer Center
Union for International Cancer Control (UICC)
United States Agency for International Development
University of Chicago Comprehensive Cancer Center
University of Colorado Cancer Center
University of Hawaii Cancer Center
University of Kansas Cancer Center
University of Maryland Marlene and Stewart Greenebaum Comprehensive Cancer Center
University of Michigan Comprehensive Cancer Center
University of New Mexico Comprehensive Cancer Center
University of Pittsburgh Cancer Institute
University of Texas MD Anderson Cancer Center
University of Wisconsin Carbone Cancer Center
USC Norris Comprehensive Cancer Center
**Organization**
Vanderbilt-Ingram Cancer Center
Wake Forest Baptist Comprehensive Cancer Center
WHO Nursing and Midwifery Community of Practice
Winship Cancer Institute of Emory University
World Health Organisation – AFRO
World Health Organisation - EURO
World Health Organisation - HQ - Department of Chronic Diseases and Health Promotion (CHP)
Yale Cancer Center

© 2025 Eldridge L et al. *JAMA Network Open*.

eTable2 – Survey responses by respondents’ country of affiliation

Country	Response N (%) (n=130)
Argentina	3 (2%)
Armenia	2 (2%)
Brazil	15 (12%)
Burundi	2 (2%)
Cameroon	1 (1%)
Chile	1 (1%)
China	5 (4%)
Congo, Rep.	1 (1%)
Costa Rica	1 (1%)
Côte d’Ivoire	2 (2%)
Cuba	1 (1%)
Egypt, Arab Rep.	1 (1%)
Estonia	1 (1%)
Ethiopia	1 (1%)
Ghana	2 (2%)
Guatemala	3 (2%)
India	17 (13%)
Japan	1 (1%)
Kenya	10 (8%)
Lebanon	3 (2%)
Malawi	2 (2%)
Malaysia	1 (1%)
Mexico	4 (3%)
Nepal	1 (1%)
Nigeria	7 (5%)
Paraguay	1 (1%)
Peru	4 (3%)
Philippines	1 (1%)
Poland	1 (1%)
Romania	2 (2%)
Rwanda	3 (2%)
Senegal	2 (2%)
Serbia	2 (2%)
Singapore	1 (1%)
South Africa	2 (2%)
Thailand	2 (2%)
Uganda	2 (2%)
United States	15 (12%)
Uruguay	1 (1%)

© 2025 Eldridge L et al. *JAMA Network Open*.

Vietnam	1 (1%)
Zimbabwe	2 (2%)

© 2025 Eldridge L et al. *JAMA Network Open*.

eTable3 – Barriers to cancer therapeutic clinical trial involvement by excluded respondents

This table shows the reported primary barriers to cancer therapeutic clinical trial involvement in LMICs by respondents that reported being clinicians without cancer therapeutic clinical trial experience, and with interest in becoming involved in clinical trials.

Barriers	N (%) (n=139) [a]
I don't have the funding	83 (60%)
I don't know who to partner with	67 (48%)
Clinical trials are not made available in my setting	55 (40%)
I don't have the proper training	46 (33%)
I don't know where to start	36 (26%)
My institution does not have the necessary infrastructure	30 (22%)
My institution does not support clinical research	16 (12%)
I don't have the time	6 (4%)
Other[b]	3 (2%)

a Percentages do not add up to 100% due to multi-select.
b Other barriers included two unspecified and one 'bias.'

© 2025 Eldridge L et al. *JAMA Network Open*.

eMethods - Survey on Cancer Therapeutic Clinical Trials in Low- and Middle-Income Countries

OMB#: 0925-0766
Expiration Date: 09/30/2026
Public reporting burden for this collection of information is estimated to average no more than 15 minutes per response, including the time for reviewing instructions, searching existing data sources, gathering and maintaining the data needed, and completing and reviewing the collection of information. An agency may not conduct or sponsor, and a person is not required to respond to, a collection of information unless it displays a currently valid OMB control number. Send comments regarding this burden estimate or any other aspect of this collection of information, including suggestions for reducing this burden to: NIH, Project Clearance Branch, 6705 Rockledge Drive, MSC 7974, Bethesda, MD 20892-7974, ATTN: PRA (0925-0766). Do not return the completed form to this address.

The National Cancer Institute (NCI) Center for Global Health is conducting a survey to help us gain a better understanding of the current landscape of cancer therapeutic clinical trials in low- and middle-income countries (LMICs). If you are or ever have been a clinician with experience designing or carrying out a cancer therapeutic clinical trial in an LMIC, we invite you to participate in this brief survey so we can learn about your thoughts and opinions regarding concrete steps that might be taken to advance clinical trials in LMICs.

This survey is anonymous and voluntary. If published, the results will be presented in an aggregated, de-identified format. There is no compensation for survey completion. All questions are optional, and you may exit the survey at any time.

For any questions, please contact ncicghclintrialsinlmic@mail.nih.gov.

Are you or have you ever been a clinician, as defined by the NCI to mean a health professional who takes care of patients?

○ Yes

○ No

Have you ever been a member of the research team for a cancer therapeutic clinical trial with at least one recruitment site/facility in a low- and middle-income country (LMIC), as defined by the World Bank?

○ Yes

○ No

Are you interested in being involved in conducting cancer therapeutic clinical trials in LMICs?

○ Yes

○ No

© 2025 Eldridge L et al. *JAMA Network Open*.

In your experience, what have been the primary barriers that have prevented you from leading or being involved in cancer therapeutic clinical trials in LMICs? Please choose no more than three.

- ☐ I don’t know where to start
- ☐ I don’t have the proper training
- ☐ I don’t have the time
- ☐ I don’t have the funding
- ☐ I don’t know who to partner with
- ☐ My institution does not have the necessary infrastructure
- ☐ My institution does not support clinical research
- ☐ Clinical trials are not made available in my setting
- ☐ Other (please specify) ____

Thank you for taking the time to complete the above questions. You do not meet our eligibility criteria for this survey. If you are aware of other individuals who meet the eligibility criteria of this survey, we invite you to share this survey link with them.

In the remainder of this survey, the term "**clinical trials**" refers to cancer therapeutic clinical trials with at least one recruitment site in an LMIC.

What is your primary specialty? Select one.

- ○ General practice
- ○ Radiotherapy
- ○ Surgery
- ○ Hematology
- ○ Gynecology-Oncology
- ○ Medical oncology

© 2025 Eldridge L et al. *JAMA Network Open*.

- ○ Clinical oncology
- ○ Nursing
- ○ Pathology
- ○ Palliative care
- ○ Pediatric oncology
- ○ Pharmacy
- ○ Radiology
- ○ Anesthesiology
- ○ Researcher
- ○ Internal medicine
- ○ Other (please specify) ____

Please select the option(s) that best represents your work setting. Select all that apply.

- ☐ Academic, such as a college or university
- ☐ Public hospital, health center, or clinic
- ☐ Private hospital, health center, or clinic
- ☐ Non-profit organization
- ☐ Industry/pharmaceutical company
- ☐ Government, such as a Ministry of Health
- ☐ Other (please specify) ____

The next series of questions will ask you to rate potential **challenges** you may have experienced in carrying out a cancer therapeutic clinical trial in an LMIC.

© 2025 Eldridge L et al. *JAMA Network Open*.

Based on your experience, please rate the following **human capacity** challenges by the impact they have had on your ability to carry out a cancer therapeutic clinical trial in an LMIC.

	No impact	Slight impact	Moderate impact	Large impact
Lack of research training	○	○	○	○
Personnel shortage	○	○	○	○
Lack of dedicated research time	○	○	○	○
Competing priorities	○	○	○	○
Lack of provider awareness of trials	○	○	○	○
Lack of mentorship	○	○	○	○
Other human capacity challenges (please specify)	○	○	○	○

© 2025 Eldridge L et al. *JAMA Network Open*.

Based on your experience, please rate the following **infrastructure and resources** challenges by the impact they've had on your ability to carry out a cancer therapeutic clinical trial in an LMIC.

	No impact	Slight impact	Moderate impact	Large impact
Lack of access to drugs or products	○	○	○	○
Equipment shortages, supply chain disruptions, or broken malfunctioning/damaged equipment	○	○	○	○
Difficulties with data management systems	○	○	○	○
Space or storage shortage	○	○	○	○
Insufficient biobanking	○	○	○	○
Insufficient diagnostics	○	○	○	○
Other infrastructure and resource challenges, (please specify)	○	○	○	○

© 2025 Eldridge L et al. *JAMA Network Open*.

Based on your experience, please rate the following **ethical and regulatory systems** challenges by the impact they've had on your ability to carry out a cancer therapeutic clinical trial in an LMIC.

	No impact	Slight impact	Moderate impact	Large impact
Decision delays	○	○	○	○
Burdensome procedures (e.g., complicated, repetitive, unclear)	○	○	○	○
Lack of trained regulatory authorities	○	○	○	○
Other ethical and regulatory systems challenges (please specify)	○	○	○	○

Based on your experience, please rate the following **financial** challenges by the impact they've had on your ability to carry out a cancer therapeutic clinical trial in an LMIC.

	No impact	Slight impact	Moderate impact	Large impact
Difficulty obtaining funding for investigator-initiated trials	○	○	○	○
Difficulty obtaining funding in general	○	○	○	○
Complex grant application/ funding process	○	○	○	○
Excessive trial costs	○	○	○	○
Lack of interest by pharmaceutical companies	○	○	○	○
Other financial challenges (please specify)	○	○	○	○

© 2025 Eldridge L et al. *JAMA Network Open*.

Based on your experience, please rate the following **administrative** challenges by the impact they've had on your ability to carry out a cancer therapeutic clinical trial in an LMIC.

	No impact	Slight impact	Moderate impact	Large impact
Lack of institutional support for research	○	○	○	○
Lack of institutional experience with trial management	○	○	○	○
Other administrative challenges (please specify)	○	○	○	○

Based on your experience, please rate the following **healthcare or sociopolitical** challenges by the impact they've had on your ability to carry out a cancer therapeutic clinical trial in an LMIC.

	No impact	Slight impact	Moderate impact	Large impact
Fragmented healthcare system	○	○	○	○
Coordination of efforts to implement cancer clinical trials	○	○	○	○
Lack of government political will	○	○	○	○
Difficulties identifying collaborators/partners	○	○	○	○
Other healthcare of sociopolitical challenges (please specify)	○	○	○	○

© 2025 Eldridge L et al. *JAMA Network Open*.

Based on your experience, please rate the following **trial design** challenges by the impact they've had on your ability to carry out a cancer therapeutic clinical trial in an LMIC.

	No impact	Slight impact	Moderate impact	Large impact
Irrelevant study questions	○	○	○	○
Inappropriate standard therapy arm	○	○	○	○
Increasing complexity of trials	○	○	○	○
Other trial design challenges (please specify)	○	○	○	○

Based on your experience, please rate the following **patient enrollment** challenges by the impact they've had on your ability to carry out a cancer therapeutic clinical trial in an LMIC.

	No impact	Slight impact	Moderate impact	Large impact
Lack of insurance coverage	○	○	○	○
Difficulties accessing care by patients (e.g., travel costs, missing work)	○	○	○	○
Restrictive eligibility	○	○	○	○
Distrust of clinical research or medical providers	○	○	○	○
Lack of clinical trials awareness	○	○	○	○
Other patient enrollment challenges (please specify)	○	○	○	○

Please comment on any of the challenges listed in the previous questions, or share other challenges that you have experienced:

© 2025 Eldridge L et al. *JAMA Network Open*.

In your opinion, what should the cancer research community prioritize to increase opportunities/capacities to conduct cancer therapeutic clinical trials in LMICs? Please rate the following strategies by level of importance.

	Not at all important	Slightly important	Moderately important	Very important	Extremely important
Build or strengthen partnerships (e.g.; regional, in-country, cross-income, etc.),	○	○	○	○	○
Build human capacity (e.g., training, protected time, etc.)	○	○	○	○	○
Create or improve ethical or regulatory systems (e.g., reform systems, train authorities)	○	○	○	○	○
Strengthen material capacity or infrastructure (e.g., access to drugs, equipment, biobanking, etc.)	○	○	○	○	○
Create a research environment (data collection and management systems, research incentives, awareness, etc.)	○	○	○	○	○
Improve funding (e.g.; funding opportunities for LMICs, simplify grant process, etc.)	○	○	○	○	○

© 2025 Eldridge L et al. *JAMA Network Open*.

Build government political will (e.g., lobby for funding, increase awareness, etc.)	○	○	○	○	○
Engage community (e.g., community gatherings, advertisements, etc.)	○	○	○	○	○

Please comment on any of the strategies in the previous question, or share other strategies that you think the cancer research community should prioritize:

____

The next two questions will ask you about the **types of clinical trials** you think a network should prioritize.

What should be the phase focus of a cancer therapeutic clinical trials network in LMICs? Select all that apply.

- ▢ Early phase development (i.e., trials evaluating safety and tolerability of a new treatment).
- ▢ Late phase approval (i.e., trials evaluating efficacy of a new treatment)
- ▢ De-escalation/efficiency/pragmatic designs (i.e., trials evaluating effectiveness in routine practice or innovative ways of delivering guideline accepted therapies)
- ▢ Other (please specify) ____

© 2025 Eldridge L et al. *JAMA Network Open*.

What should be the geographic focus of a cancer therapeutic trials network in LMICs?

- ○ Multinational trials with high income country (HIC) **and** LMIC sites
- ○ Multinational trials with LMIC sites **only**
- ○ Trials restricted to **one** LMIC
- ○ Other (please specify) ____

The next series of questions will you ask you about your experience with **cancer therapeutic clinical trials** in LMICs.

What has been your level of involvement in clinical trials? Select all that apply.

- ☐ Principal Investigator (PI), individual responsible for scientific and technical direction of study (e.g., concept development, protocol writing, data collection supervision, etc.)
- ☐ Site Principal Investigator (PI), individual responsible for the conduct of a clinical study at a site, but not responsible for study design (e.g., site activation, recruitment, etc.)
- ☐ Co-investigator, individual who makes substantial contributions to a clinical study, but who does not have the overall responsibility and authority of the PI (e.g., data collection, analysis, or interpretation, etc.)

Other member of research team (please specify) ____

© 2025 Eldridge L et al. *JAMA Network Open*.

Which trial phase represents most of the clinical trials you've been involved in? Select one.

- ○ Phase 1, to evaluate safety and identify side effects
- ○ Phase 2, to determine effectiveness and evaluate safety
- ○ Phase 3, to confirm its effectiveness, monitor side effects, compare it with standard or equivalent treatments
- ○ Phase 4 (post-market), to seek more information about its risks, benefits, and optimal use following approval as well as learning more about side effects, especially rare side effects

Which cancer site(s) represents most of the clinical trials you've been involved in? Select all that apply.

- ☐ Breast
- ☐ Cervical
- ☐ Colorectal
- ☐ Esophageal
- ☐ HIV-associated
- ☐ Leukemia
- ☐ Lip, oral cavity
- ☐ Liver
- ☐ Lung
- ☐ Lymphoma
- ☐ Pancreas
- ☐ Pediatric
- ☐ Prostate

© 2025 Eldridge L et al. *JAMA Network Open*.

☐ Stomach

☐ Other (please specify) ____

Among the clinical trials you have been involved in, approximately what percentage recruited **most or all** participants in LMICs? Select one.

○ Less than 25% of trials recruited most or all participants from a facility in an LMIC

○ 25-50% of trials recruited most or all participants from a facility in an LMIC

○ 51-75% of trials recruited most or all participants from a facility in an LMIC

○ 76-100% of trials recruited most or all participants from a facility in an LMIC

○ Unsure/I don't know

What percentage of the clinical trials you've been involved with have received **funding** (all or in part) from each category:

	None	1-25%	26-50%	51-75%	76-100%
Industry or pharmaceutical	○	○	○	○	○
Governmental organization	○	○	○	○	○
Academic institution:	○	○	○	○	○
Other (please specify)	○	○	○	○	○

© 2025 Eldridge L et al. *JAMA Network Open*.

The following questions about your **identity and background** will be kept private. Responses are anonymous and will be reported in aggregate. Your responses will be used to better understand our results.

Are you: (Select all that apply)

- ☐ Female
- ☐ Male
- ☐ Transgender, non-binary, or another gender
- ☐ Decline to answer

Please select your current career stage:

- ○ Student
- ○ Trainee (fellow, resident)
- ○ Early-career (completed terminal degree or post-graduate training within the past 10 years)
- ○ Mid-career (10-20 years)
- ○ Late-career (≥20 years)
- ○ Other (please specify) ____

Please share your primary country of **residence**: (Primary residence is where you normally spend most of your days in one year.)

▼ Afghanistan ... Zimbabwe

Please share the country of your primary **institutional** affiliation:

▼ Afghanistan ... Zimbabwe

Please provide any additional comments:

____

© 2025 Eldridge L et al. *JAMA Network Open*.

This survey is anonymous; however, if you agree to be contacted for further comment related to this area, please provide your name and email address. This is entirely optional, and your responses will not be connected to you in any way.

- ○ First/given name: ____________________
- ○ Last/Surname: ____________________
- ○ Email address: ____________________

© 2025 Eldridge L et al. *JAMA Network Open*.
