# Supplementary material for: Barriers and Opportunities for Cancer Clinical Trials in Low- and Middle-Income Countries
Source: JAMA Netw Open. 2025 Apr 28;8(4):e257733. doi: 10.1001/jamanetworkopen.2025.7733 (PMC12038506; doi:10.1001/jamanetworkopen.2025.7733)
Supplement: Supplement 2. — Data Sharing Statement [file jamanetwopen-e257733-s002.pdf]

# Data Sharing Statement

Eldridge. Barriers and Opportunities for Cancer Clinical Trials in Low- and Middle-Income Countries. *JAMA Netw Open*. Published April 28, 2025.
doi:10.1001/jamanetworkopen.2025.7733

**Data**
**Data available:** No
